# At Your Leisure

**Published:** 1896-03

**Authors:** 


### AT YOUR LEISURE.

The Philadelphia *Times* of January 9th had an illustrated article descriptive of the Veterinary Department of the University of Pennsylvania. The illustrations of the hospital, dissecting-rooms, and operating sections were excellent, and, accompanied by an interesting history of the growth of the Department, made interesting reading.

The experiments at the Cornell Experiment Station, conducted upon two series of animals, to determine the value of fat, fed in the shape of tallow, for the purpose of increasing the butter-fat in the milk, proved only the fallacy of the theory and proposition as urged by others.

The February meeting of the Keystone Veterinary Medical Association was the most interesting and instructive local meeting held for many years in Philadelphia. The programme was contributed to by Drs. Eves, McAnulty, McBirney, and Underhill.

The importance of the swine-breeding industry is attested by a sale of breeding-sows at Shenandoah, Iowa, when a single sow sold for $505, and forty-six sows averaged $90 each; worth more than horses.

The National Cat-Show will be held this year at Madison Square Garden, New York City, March 3 to 7, 1896.

Out of some three operations recently performed in the western part of Pennsylvania for roaring, but one proved beneficial.

Up to the date of June 30, 1894, thirty-one veterinarians underwent the civil-service examination for Assistant Meat-Inspectors in the Bureau of Animal Industry. Of this number three passed, but none were appointed. For the year ending June 30, 1895, ninety-four presented themselves for examination, thirty-five of whom passed, fourteen of whom have been appointed to positions at salaries ranging from $1200 to $1400 per year. The Chief of the Bureau of Animal Industry receives a salary of $4000 per year.

An extract of passiflora incarnata (passion flowers) is strongly recommended for use in tetanus to control the spasms; preferably in connection with cannabis indica.

The graduation exercises of the New York College of Veterinary Surgeons will take place on April 1st, and will be an innovation on the long-established custom of having public exercises. The diplomas will be awarded in conjunction with a dinner given by the faculty, alumni, and graduating-class.

Professor R. S. Huidekoper has placed in the hands of his publisher the specimen pages of an illustrated and descriptive book on horseshoeing. The work promises to become a very complete and valuable contribution to the literature on this subject to the veterinarian, master horseshoer, breeders, and owners of valuable horses.

The closing exercises of the American Veterinary College will take place March 28, 1896. The alumni meeting will be held at 2.30 P.M. on the afternoon of commencement day at the College Hall; the closing exercises at Chickering Hall in the evening.

Dehorning of cattle in Salem County, N. J., has become very popular, and ere long most herds will be freed from these weapons of offense and defense.

Virginia possesses to-day one of the most active and aggressive veterinary associations in our land. Their meetings are well attended and the programme is always loaded down with a grand intellectual feast. She is knocking at the door of the State Legislature for better safeguards to those coming to the

State properly equipped to practise veterinary science, and her people will do well to grant her reasonable demands, which after all contribute more to the benefit of her people than can possibly accrue to the veterinarians.

The bill introduced in Congress by Senator Sherman looking to an increase of the service to twelve companies to a regiment, so that each regiment may consist of three battalions of four companies to each battalion, provides for the appointment of veterinarians to cavalry and artillery, which will increase the number of veterinarians now required.

The annual holiday edition of the Alameda *Argus*, of Alameda, Cal., gives an interesting account of the milk-inspection departments of the Board of Health. The earliest investigation in the State of bovine tuberculosis with tuberculin has been made. By this Board of Health, under the direct supervision of T. Carpenter, veterinary inspector, all milk-producers and vendors for the city are required to take out licenses.

The death of Lieutenant M. H. Doe, of the Police Department of Lynn, Mass., after an unsuccessful trial of electricity to the extent of 5000 volts, gives little promise of the value of this remedy in this much-dreaded disease.

Ohio and Illinois have laws that limit the sale of all drugs and medicines to pharmacists, and hold the latter individually responsible for the purity and standard strength of everything they sell.

